# Modified Gap Arthroplasty for Temporomandibular Joint Ankylosis Following Radiotherapy for Rhabdomyosarcoma: Report of an Unusual Case and Brief Literature Review

**DOI:** 10.3389/fonc.2021.784690

**Published:** 2021-11-26

**Authors:** Jianfei Zhang, Liyan Dai, Ahmed Abdelrehem, Jinyang Wu, Xiaobo Li, Steve Guofang Shen

**Affiliations:** ^1^ Department of Oral & Cranio-maxillofacial Surgery, Shanghai Ninth People’s Hospital, College of Stomatology, National Clinical Research Center for Oral Diseases, Shanghai Key Laboratory of Stomatology & Shanghai Research Institute of Stomatology, Shanghai Jiao Tong University School of Medicine, Shanghai, China; ^2^ Department of Radiation Oncology, Renji Hospital Affiliated to Shanghai Jiao Tong University School of Medicine, Pudong Shanghai, China; ^3^ Department of Craniomaxillofacial and Plastic Surgery, Faculty of Dentistry, Alexandria University, Alexandria, Egypt; ^4^ Department of Radiation Oncology, Fujian Medical University Union Hospital, College of Medical Technology and Engineering, Fujian Medical University, Fuzhou, China

**Keywords:** gap arthroplasty, TMJ ankylosis, radiotherapy, osteoradionecrosis, rhabdomyosarcoma

## Abstract

Radiotherapy at the temporomandibular joint (TMJ) area often results in trismus, however, post radiation ankylosis is extremely rare and has not been previously reported in literature. Radiation is known to impact the vasculature of bony structures leading to bone necrosis with certain risk factors including surgical intervention, even teeth extraction, that could lead to osteoradionecrosis. Accordingly, gap arthroplasty for such case seemed rather challenging. In this report, we introduce for the first time, a rare case of temporomandibular joint ankylosis post radiotherapy for management of rhabdomyosarcoma in a 12 years-old boy. A modified gap arthroplasty technique combined simultaneously with pterygo-masseteric muscle flap was applied to lower the risk of osteoradionecrosis due surgical trauma at irradiated area. Computed tomographic scan on the head indicated that the TMJ architecture was completely replaced by bone, with fusion of the condyle, sigmoid notch, and coronoid process to the zygomatic arch and glenoid fossa. The patient’s problem was totally solved with no osteoradionecrosis or relapse of ankylosis observed at follow up visits. Herein, the modified gap arthroplasty combined with pterygo-masseteric muscle flap could be recommended to be applied on other cases of ankylosis especially after receiving radiotherapy.

## Introduction

Radiotherapy for the management of head and neck cancers often impacts the temporomandibular joint (TMJ) and masticatory muscles due to the close proximity of the target region and the TMJ (1–3). This usually results in joint dysfunction such as trismus, which is regarded as the most common late complication of radiation on the TMJ area (4, 5). Although there are many published studies of trismus following radiotherapy, a review of the English language literature revealed no studies reporting ankylosis of the TMJ following radiotherapy for the management of head and neck cancers. More importantly, treatment of radiation-induced ankylosis is challenging due to a lack of understanding of this condition, its rarity, and its irreversibility.

The present work describes an extremely rare case of post-radiation TMJ ankylosis in a 19-year-old patient who was diagnosed with rhabdomyosarcoma at the age of 12. Here, the emphasis is on treatment, which was challenging in this case due to the risk of inducing osteoradionecrosis following conventional surgical techniques. Therefore, a modified gap arthroplasty technique combined with a pterygo-masseteric muscle flap as an interpositional material was applied for management of the irradiated ankylosed TMJ, in order to avoid postoperative bone necrosis and achieve optimum mouth opening (6).

## Case Report

**Figure 1:**
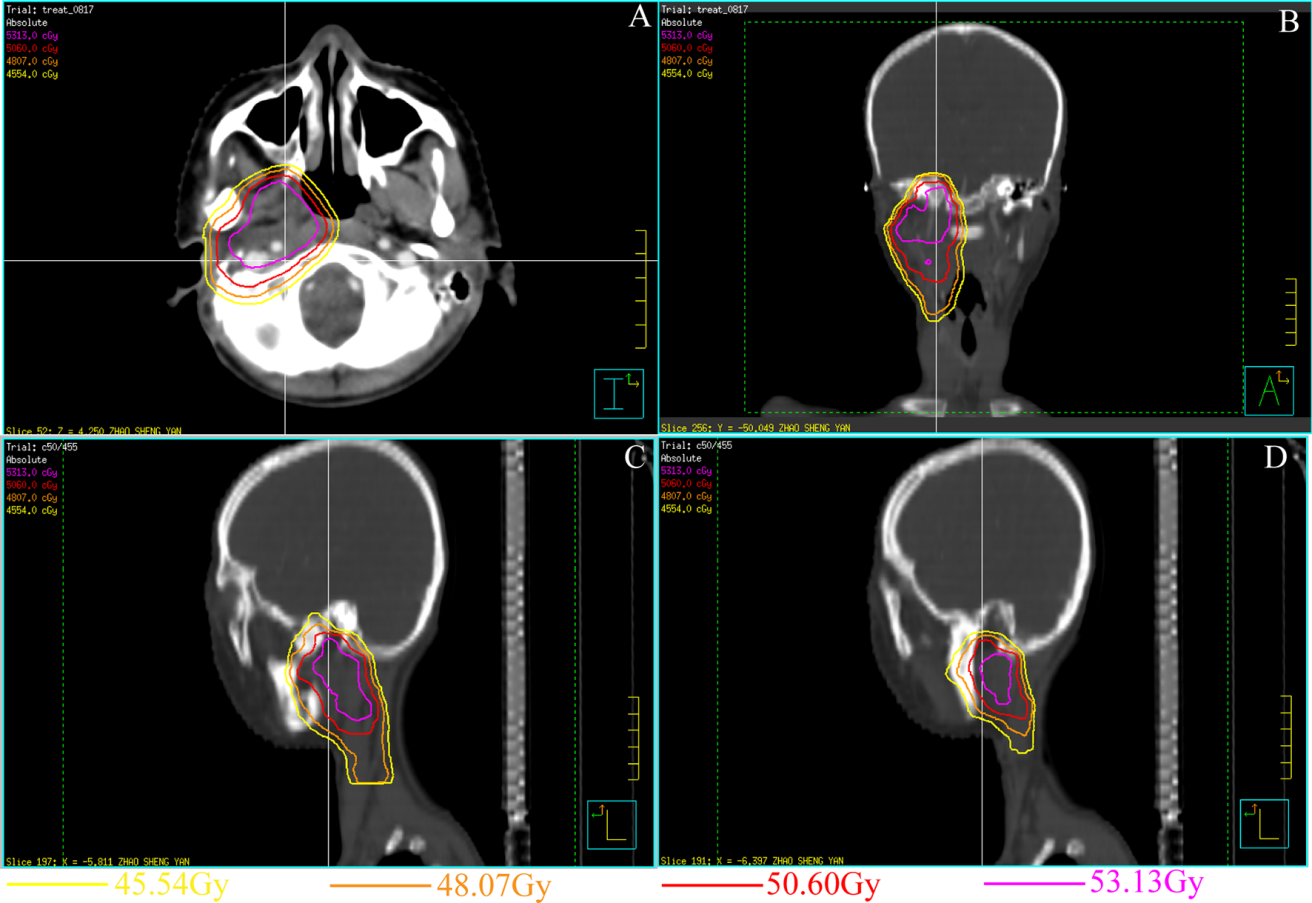
Treatment planning and set-up images for the rhabdomyosarcoma in the right pterygoid muscle 7 years ago **(A–D)**. **(A)** Horizontal view CT image of the radiation area. **(B)** Coronal view CT image of the radiation area. The inner side of the right condylar area was exposed to high-dose radiation. **(C, D)** Sagittal view CT image of the radiation area.

A 19-year-old male patient presented to our department with the chief complaint of an inability to open his mouth after receiving multiple sessions of radiotherapy (59 sessions) following a diagnosis of rhabdomyosarcoma in the right infratemporal region at another hospital 7 years earlier. The patient reported progressively limited mouth opening over the preceding six months following the completion of radiotherapy, with a total radiotherapy dose of 50.64 Gy (**Figures 1A–D**). At presentation to hospital, he was no longer able to open his mouth at all.

**Figure 2:**
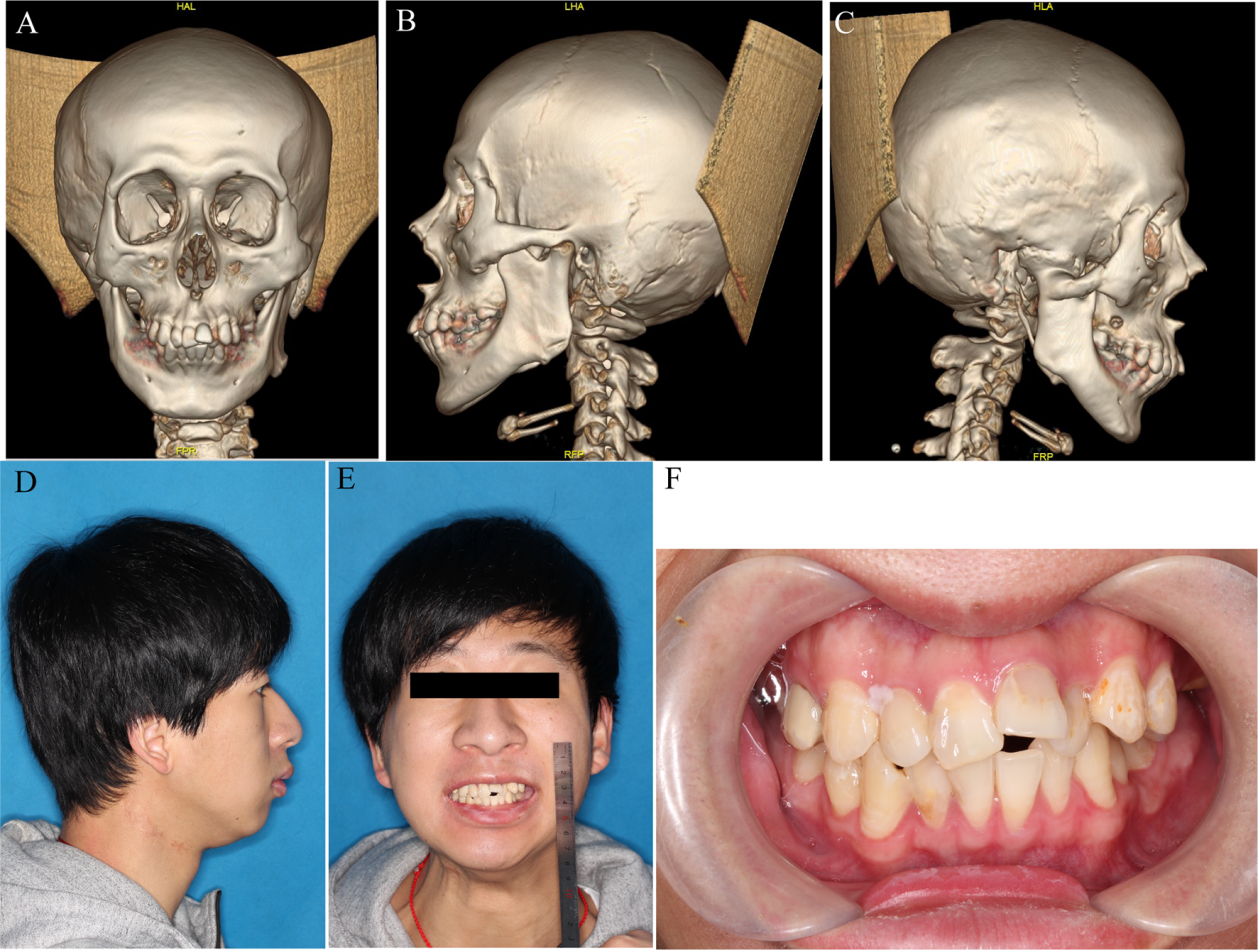
Pre-operative profile. **(A–C)** 3D reconstruction of bony ankylosis of right TMJ. **(D, E)** Lateral and front view before surgery. **(F)** Occlusal relationship before surgery.

3D reconstruction of computed tomography (CT) scans of the head and neck region showed that the right condyle was completely fused to the glenoid fossa by bony ankylosis (**Figures 2A** **–F**). Some bone necrosis could be observed around the right bony condyle in the horizontal sections of the CT images; therefore, an increased risk of bony necrosis after gap arthroplasty was anticipated and prudently considered.

To decrease the risk of bony necrosis after surgery, a modified gap arthroplasty procedure was designed to avoid the irradiated area. In addition, a 3D-printed guide was used to help perform the osteotomy precisely.

**Figure 3:**
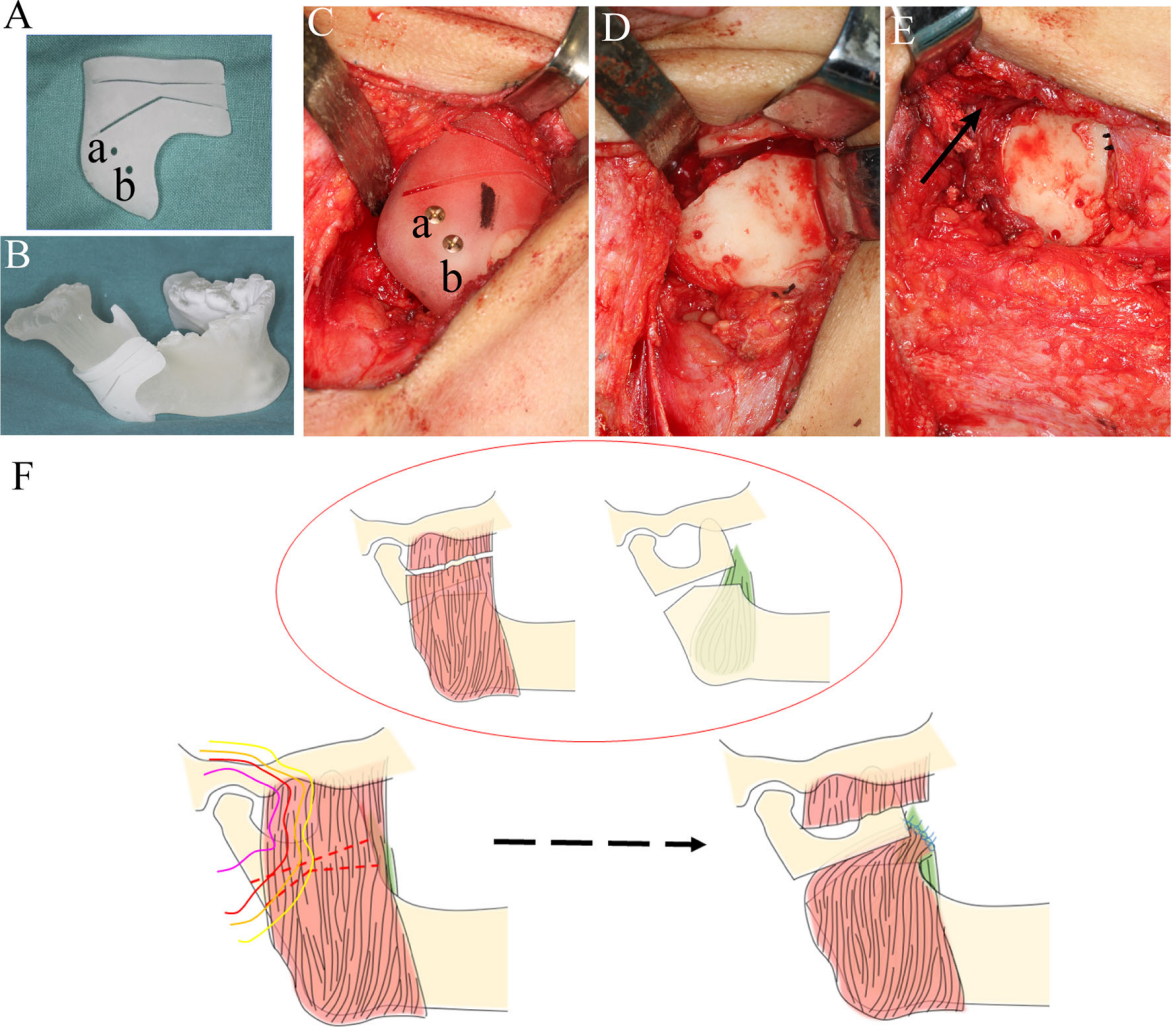
Surgical procedure and graphic presentation. **(A)** A 3D-printed custom guide was fabricated to confirm the osteotomy plane. **(B)** A 3D-printed custom mandible was fabricated. Two drills were designed (a, b) to fix the guide **(B, C)**. **(C)** The guide was fixed on the right mandibular angle. **(D)** Osteotomy was performed according to the design, and a 0.5 cm gap was made. **(E)** A pterygo-masseteric muscle flap was used as an inter-positional material (black arrow). **(F)** Sketh of the procedures of modified arthroplasty, and the colors of the lines were consistent with radiation doses in **Figure 1**.

Before surgery, the irradiated area was mapped chronographically using different colored lines to emphasize the different risk zones reflecting varying radiation doses (determined through the patient’s files). This was to ensure appropriate low-risk placement of the osteotomy line for gap arthroplasty, enabling it to be as close as possible to the right condyle to ensure enough blood supply (**Figures 1A** **–D**). Next, virtual osteotomy was performed using Proplan 3.0 (Materialise, Belgium); a custom 3D-printed surgical guide was designed and printed before surgery (**Figures 3A, B**).

Intraoperatively, the lower right mandibular ramus was exposed through a low submandibular incision at the level of the lower neck crease. Then, osteotomy was performed with the guidance of the 3D-printed template, which was fixed on the right mandibular ramus with two titanium screws (**Figure 3C**). An osteotomy was performed according to the pre-surgery plan and a 5 mm gap was made (**Figure 3D**). The masseter muscle was separated and sutured across the gap to the medial pterygoid muscle with a 3-0 absorbable suture (VICRYL Rapide, America) (**Figures 3E, F**). Meanwhile, the left coronoid process was also removed for better late outcomes (**Figures 2C** **–E**). By the end of the osteotomy procedure, the patient’s mouth was able to open 3 cm with the assistance of a mouth gag. A dental arch splint was used postoperatively for fixation of the elastic bands to obtain a more stable occlusal relationship.

**Figure 4:**
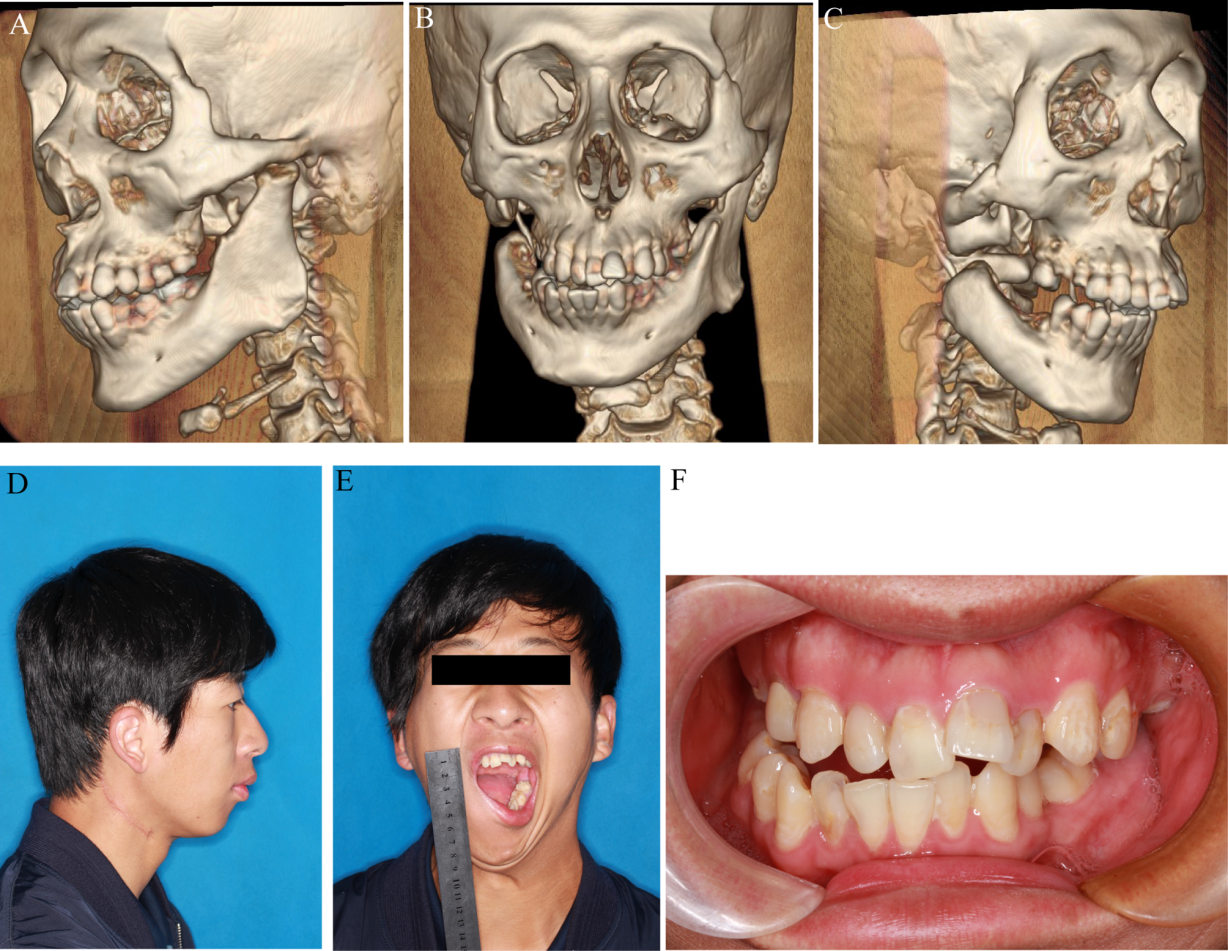
Pre-operative profile. **(A–C)** 3D reconstruction after surgery. **(D, E)** Lateral and front view before surgery. **(F)** Occlusal relationship before surgery.

The patient began jaw exercises 48 h postoperatively and these were carried out vigorously to the patient’s limit of tolerance. The discharge instructions involved the use of the mouth gag to open the mouth 5-8 times per day with each minor sprocket setting lasting a minimum of 10 min. Interestingly, the patient’s maximum mouth opening significantly increased from 0 mm to 43 mm within 6 months of surgery, and stable occlusion was maintained. Endodontic and orthodontic treatments were later performed. At the 2-year postoperative follow-up, no bone necrosis or recurrence of restricted opening was observed, with the patient reporting satisfaction with the surgical outcome (**Figures 4A–F**).

## Discussion

Radiotherapy is one of the primary treatment approaches for the management of head and neck rhabdomyosarcoma in children due to its high radiosensitivity (7). Although advances in radiotherapy for rhabdomyosarcomas have enabled promising patient outcomes, complications associated with the structures in close proximity to the irradiation areas are inevitable; in particular, the TMJ is often impacted (8). The pathogenesis of radiation complications involves varied mechanisms, including DNA damage, angiogenesis, inflammation, and tissue remodeling.

Radiation-induced trismus, resulting from damage to the TMJ, is one of the common late complications that occur following radical radiotherapy for rhabdomyosarcoma (9). Limited jaw opening is reported in 6-86% of patients who have received radiation therapy near the TMJ area, with a frequency and severity that are somewhat unpredictable (10). Nevertheless, no previous studies have described ankylosis as a result of radiation therapy for the management of head and neck rhabdomyosarcoma. Being an advanced end-stage of TMJ trismus, ankylosis affects normal mastication, speech, oral hygiene, and normal life activities, and can even be potentially life-threatening. Many surgical treatment modalities have been described for the management of TMJ ankylosis, including interpositional arthroplasty, total joint reconstruction (TJR), and gap arthroplasty (11). However, given that the ankylosis is in an irradiated region, conventional gap arthroplasty is not the optimum choice due to the higher probability of bone necrosis.

Osteoradionecrosis (ORN) is defined as non-healing, exposed bone in a previously radiated area of the body (12). ORN occurs when a bone in an irradiated field undergoes necrosis and eventually becomes exposed through the overlying soft tissues (13). Of all bones in the head and neck area, the mandible is the most common site of ORN due to its superficial location and lower blood supply. The exact incidence of mandibular ORN is still unknown, with estimates ranging from 2 to 35%. Many risk factors have been considered in the literature, including dental status, extraction after radiation therapy, mandibular surgery, total radiation dose, dose rate, and dose per fraction (14). Accordingly, any further surgical procedures in the radiation area should be carefully considered and undertaken with great caution.

Patients treated with head and neck radiation have an increased risk of development of ORN of the jaw if they undergo surgical manipulation of the jaw, particularly after radiation treatment; even the extraction of a tooth increases the risk of ORN (15). For this patient, osteoradionecrosis could have been triggered by the conventional gap arthroplasty procedure. Therefore, a modified gap arthroplasty procedure was performed over two sessions. First, the osteotomy planes were modified to avoid the main radiotherapy area. Second, a pterygo-masseteric muscle flap was used as an interpositional material instead of the pedicled buccal fat pad or abdominal fat in order to achieve better blood supply. Moreover, the suturing of the pterygo-masseteric muscle flap decreased the occlusal force on the right side, which decreased the possibility of re-ankylosis (16).

Thus, for patients with ankylosis after radiotherapy, regular arthroplasty should be considered prudentially due to the higher possibility of bone necrosis. As such, for TMJ ankylosis patients caused by radiotherapy, two important factors should be considered: the site of the osteotomy plane and the blood supply of the adjacent area.

## Conclusions

This study has described a case of right TMJ ankylosis which occurred over 6 years following exposure to radiotherapy. TMJ ankylosis can interfere with the patient’s mastication and normal life activities, and can even be life-threatening in some cases (17). After considering the standard procedures and the risks of re-ankylosis and osteoradionecrosis following arthroplasty, a modified gap arthroplasty procedure was designed and implemented with the help of a custom computer-aided design and 3D-printed guide. After a careful postoperative protocol and regular follow-ups, satisfactory outcomes were obtained. These surgical procedures can also be applied to other TMJ ankylosis patients.

## Data Availability Statement

The original contributions presented in the study are included in the article/supplementary material. Further inquiries can be directed to the corresponding authors.

## Ethics Statement

The studies involving human participants were reviewed and approved by the Medical Ethics Committee of Shanghai Ninth People’s Hospital, affiliated to Shanghai Jiaotong University School of Medicine. The patients/participants provided their written informed consent to participate in this study. Written informed consent was obtained from the individual(s) for the publication of any potentially identifiable images or data included in this article.

## Author Contributions

JZ and LD conceived of the work. JZ and AA collected all of these data. JZ and JW wrote the manuscript with support from XL and SS performed the gap arthroplasty and helped supervise the project. All authors made effort to the discussion contributed to the final manuscript.

## Funding

This project was sponsored by the “Seed Founding of Shanghai Ninth People’s Hospital, Shanghai Jiao Tong university School of Medicine (project number JYZZ087B)”, “Interdisciplinary Program of Shanghai Jiao Tong University (project number YG2021QN78).

## Conflict of Interest

The authors declare that the research was conducted in the absence of any commercial or financial relationships that could be construed as a potential conflict of interest.

## Publisher’s Note

All claims expressed in this article are solely those of the authors and do not necessarily represent those of their affiliated organizations, or those of the publisher, the editors and the reviewers. Any product that may be evaluated in this article, or claim that may be made by its manufacturer, is not guaranteed or endorsed by the publisher.
